# Mitochondrial function in intestinal epithelium homeostasis and modulation in diet-induced obesity

**DOI:** 10.1016/j.molmet.2022.101546

**Published:** 2022-07-08

**Authors:** Thomas Guerbette, Gaëlle Boudry, Annaïg Lan

**Affiliations:** 1 Institut Numecan, INSERM, INRAE, Univ Rennes, Rennes, France; 2 Université Paris-Saclay, AgroParisTech, INRAE, UMR PNCA, Paris, France

**Keywords:** Obesity, Intestine, Mitochondria, Energy metabolism, High fat diet

## Abstract

**Background:**

Systemic low-grade inflammation observed in diet-induced obesity has been associated with dysbiosis and disturbance of intestinal homeostasis. This latter relies on an efficient epithelial barrier and coordinated intestinal epithelial cell (IEC) renewal that are supported by their mitochondrial function. However, IEC mitochondrial function might be impaired by high fat diet (HFD) consumption, notably through gut-derived metabolite production and fatty acids, that may act as metabolic perturbators of IEC.

**Scope of review:**

This review presents the current general knowledge on mitochondria, before focusing on IEC mitochondrial function and its role in the control of intestinal homeostasis, and featuring the known effects of nutrients and metabolites, originating from the diet or gut bacterial metabolism, on IEC mitochondrial function. It then summarizes the impact of HFD on mitochondrial function in IEC of both small intestine and colon and discusses the possible link between mitochondrial dysfunction and altered intestinal homeostasis in diet-induced obesity.

**Major conclusions:**

HFD consumption provokes a metabolic shift toward fatty acid β-oxidation in the small intestine epithelial cells and impairs colonocyte mitochondrial function, possibly through downstream consequences of excessive fatty acid β-oxidation and/or the presence of deleterious metabolites produced by the gut microbiota. Decreased levels of ATP and concomitant O_2_ leaks into the intestinal lumen could explain the alterations of intestinal epithelium dynamics, barrier disruption and dysbiosis that contribute to the loss of epithelial homeostasis in diet-induced obesity. However, the effect of HFD on IEC mitochondrial function in the small intestine remains unknown and the precise mechanisms by which HFD induces mitochondrial dysfunction in the colon have not been elucidated so far.

## Abbreviations

CAT: Catalase
CLD: Cytosolic lipid droplet
CPT1: Carnitine palmitoyltransferase 1
DRP1: Dynamin-related protein 1
ER: Endoplasmic reticulum
ETC: Electron transport chain
FADH: Flavin adenine dinucleotide
GO_BP: Gene ontology biological process
GPX: Glutathione peroxidase
GTP: Guanosine triphosphate
HDAC: Histone deacetylase
HFD: High fat diet
HK1: Hexokinase 1
HIF1α: Hypoxia-inducible factor 1α
HMGCS2: 3-hydroxy-3-methylglutaryl-CoA synthase 2
HSP: Heat shock protein
IEC: Intestinal epithelial cells
IMM: Inner mitochondrial membrane
IMS: Intermembrane space
ISC: Intestinal stem cells
LGR5: Leucine-rich repeat-containing G-protein coupled receptor 5
MFN: Mitofusin
Miro: Mitochondria Rho-GTPase
mt: Mitochondria
mTOR: Mitochondrial target of rapamycin
NAD: Nicotinamide adenine dinucleotide
NRF: Nuclear respiratory factor
OMM: Outer mitochondrial membrane
OXPHOS: Oxidative phosphorylation
Parkin: RBR E3 Ubiquitin Protein Ligase
PGC1: Peroxisome proliferator activated receptor γ coactivator-1
PINK1: Phosphatase and tensin homolog-induced kinase 1
PPAR: Peroxisome proliferator-activated receptor
PUMA: p53 upregulated modulator of apoptosis
ROS: Reactive oxygen species
SCFA: Shortchain fatty acids
SIRT: Sirtuin
SOD: Superoxide dismutase
TCA: Tricarboxylic acid cycle

## 1 Introduction

Overweight and obesity are defined as abnormal or excessive fat accumulation that presents a risk to health owing to an energy imbalance between calories consumed and calories expended. According to recent reports from the World Health Organization (WHO), 1.9 billion adults were overweight and 650 million obese in 2016. Four million overweight or obese people die each year from associated complications, such as cardiovascular diseases or cancer [1]. Those obesity-associated metabolic disorders are likely to occur as a result of systemic low-grade inflammation [2,3]. Indeed, because of the major endocrine function of adipose tissue notably in adipokine production, excessive fat accumulation in obese subjects leads to metabolic inflammation of numerous tissues such as liver, muscle or brain [4,5]. However, disturbances of intestinal homeostasis, in particular an increased permeability of the intestinal barrier, concomitant with intestinal microbiota alterations of its composition and/or metabolic activities, as described in animal model [[6], [7], [8]] as well as in humans [9], also participate in the onset and/or perpetuation of systemic low-grade inflammation [10]. Intestinal homeostasis relies on complex interactions between the microbiota, the intestinal epithelium, and the host immune system, that allow the intestinal barrier function maintenance. This barrier is notably made of a monolayer of intestinal epithelial cells (IEC), associated with each other by tight junctions, and a mucus layer that protects the IEC surface. In physiological situations, the intestinal barrier acts as a filter that absorbs nutrients in the small intestine and water and electrolytes in the colon. Those absorption processes involve transporters that require high amounts of energy. In addition, the intestinal epithelium constantly renews itself every 4–5 days. This renewal is ensured by intestinal stem cells (ISC), nested at the bottom of intestinal crypts, which undergo continuous asymmetric division generating progenitor cells that differentiate as they migrate up the crypt-villus axis in the small intestine, or up the crypt in the colon, and finally die by anoikis. A balance between proliferation/apoptosis contributes to intestinal homeostasis whereas disruption of this equilibrium and increased apoptosis is associated with defects of the intestinal barrier [11].

Because of the energy needed for rapid epithelial turnover, ATPase-dependent transporters, and junctional cell permeability regulations as well as mucus glycoproteins and antimicrobial peptides synthesis, the intestinal epithelium requires great amounts of energy. Henceforth, the gastrointestinal tract represents only 5% of the total body weight, but consumes 20% of the whole-body oxygen [12]. Energy is produced by mitochondria, in the form of ATP via oxidative phosphorylation (OXPHOS). Mitochondrial function plays therefore a pivotal role in intestinal homeostasis. However, IEC mitochondrial function is likely to be impaired by high fat diet (HFD) consumption notably through nutrients, such as fatty acids, or metabolites produced by the gut microbiota, that may act as metabolic perturbators of IEC. Mitochondrial dysfunction is thus defined as any mechanism that reduces efficiency of OXPHOS and leads to decreased levels of cellular ATP.

The objectives of this review are first to resume the current knowledge on mitochondria, then focus on mitochondrial function in IEC and in the control of intestinal homeostasis and highlight the known effects of metabolites, originating from the diet or gut bacterial metabolism, on IEC mitochondrial function. The final objective is to summarize the current literature on the impact of HFD on mitochondrial function in IEC and discusses the possible link between mitochondrial dysfunction and altered intestinal homeostasis in diet-induced obesity.

## 2 General description of mitochondria

### 2.1 Structure

**Figure 1:**
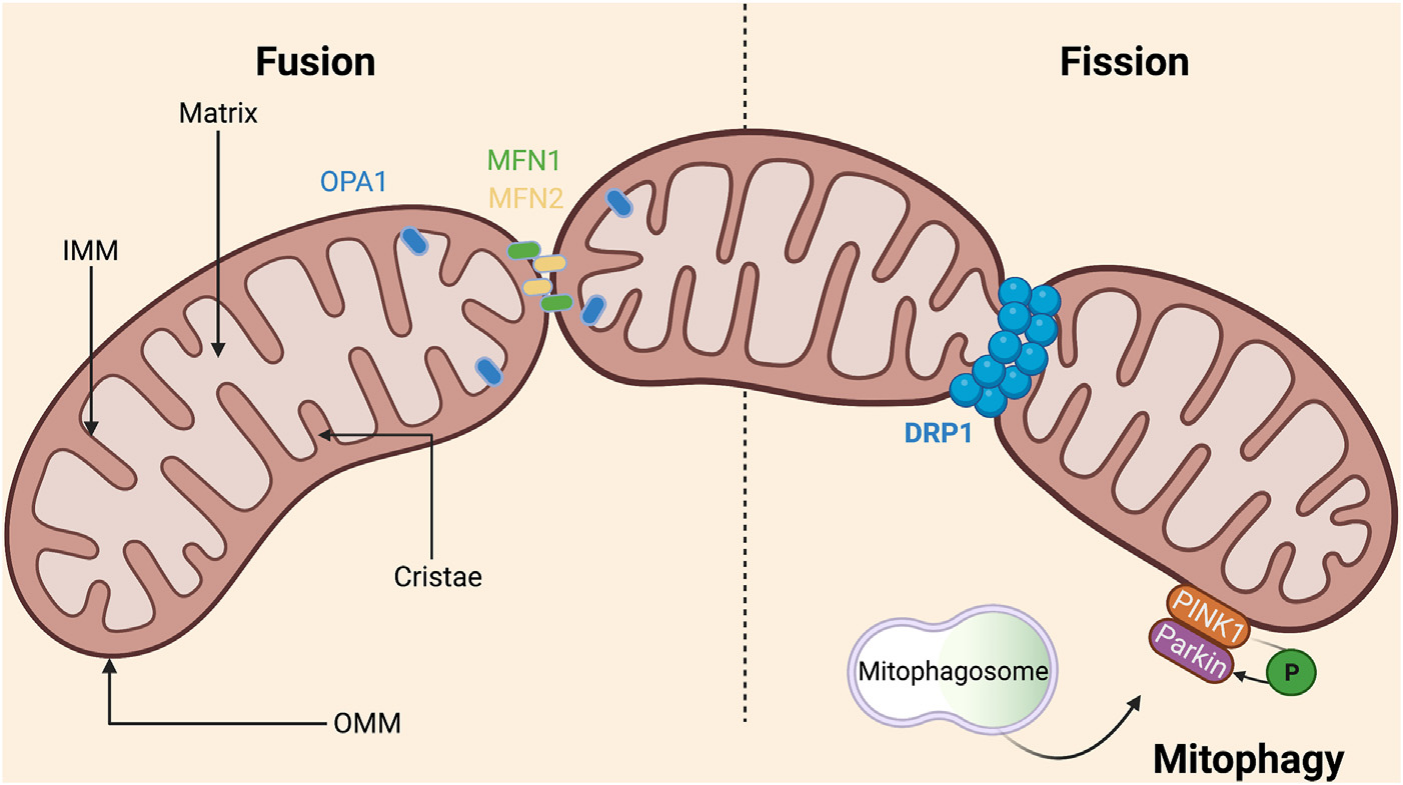
**Mitochondrial structure and dynamics.** This schematic representation of mitochondrial architecture shows inner mitochondrial membrane (**IMM**) folded in cristae, which houses the mitochondrial electron transport chain, and surrounds the mitochondrial matrix. Outer mitochondrial membrane (**OMM**) separates the intermembrane space from the cytosol. Mitochondrial dynamics involves **fusion** and **fission**. Fusion of two mitochondria into a bigger one is notably mediated in the OMM by mitofusin **(MFN) 1** and **MFN2** isoforms, which form homo or heterodimers, and by optic atrophy 1 (**OPA1**) in IMM which facilitates inner membrane merging. At the opposite, mitochondrial **fission** provides smaller mitochondria from a bigger one and occurs where endoplasmic reticulum (ER) makes contacts with mitochondria, creating the fission apparatus composed of OMM protein, such as mitochondrial dynamics proteins of 49 and 51 kDa, and GTPase Dynamin-related protein 1 (**DRP1**) on sites where ER attach. DRP1 then translocates to the OMM, multimerizes, forming a ring structure around the organelle which finally splits both membranes. Once mitochondrial fission is completed, the healthy mitochondrion is reintegrated to the tubular network whereas the downregulation of fusion mediators isolates damaged mitochondria. Damaged mitochondria, are characterized by the phosphatase and tensin homolog-induced kinase 1 (**PINK1**) accumulation on the OMM and are recognized by the cytosolic RBR E3 ubiquitin protein ligase (Parkin) that phosphorylates PINK1, triggering OMM protein ubiquitination and recruiting mitophagasomes machinery. The phagosome fuses with a lysosome and proceeds to mitochondrial degradation via lysosomal proteases and lipases to finally eliminate damaged mitochondria by mitophagy. (For interpretation of the references to colour in this figure legend, the reader is referred to the Web version of this article.)

Mitochondria are 0.5–1 μm long by 0.5–1 μm large organelles that are delimited by two phospholipid bilayers: an inner mitochondrial membrane (IMM) and an outer mitochondrial membrane (OMM), separated by an intermembrane space (IMS) (Figure 1). The OMM separates the mitochondrial content from the cytosolic space and allows the communication of mitochondria with its environment, notably through mitochondria-associated membranes (MAM) which permit functional and physical communication between mitochondria and the endoplasmic reticulum (ER). The OMM also constitutes an exchange interface between the mitochondria and the cytosol. While proteins and ions smaller than 5 kDa diffuse through the OMM via porins, bigger molecules, such as pre-proteins which possess a mitochondrial targeting signal, translocate across the OMM via binding to translocases of OMM complex [13]. Proteins internalized into the mitochondria include heat shock proteins (HSP) or proteins involved in the tricarboxylic acid cycle (TCA), β-oxidation and/or OXPHOS. On the other hand, the IMM is an impermeable membrane that allows lipid trafficking. It houses the electron transport chain (ETC) complexes, involved in ATP production via OXPHOS. Inward folds, called cristae, extend IMM thus increasing surface for ATP generation and facilitating protein and metabolite exchanges through IMM translocases [16].

Mitochondria are also mobile organelles able to move along microtubules via a mitochondrial Rho-GTPase (Miro), which anchors kinesin motor to the mitochondrial surface, forming a dynamic network [17].

### 2.2 Mitochondrial genome

Mitochondria possess their own genome, a circular double-stranded mtDNA (∼16.6 kb in humans) housed inside the matrix. Mitochondrial genome is made of hundreds to thousands of mtDNA copies per cell depending on the tissue [18], only transmitted maternally, although this has been recently challenged [19]. Strong evidences suggest that the number of mitochondria and mtDNA copies is function of the cellular energy requirements since high energy demands tend to require an enhanced number of mitochondria and more copies of mtDNA than low energy demand [20].

### 2.3 Mitochondrial dynamics: biogenesis, fission, fusion, and control quality

#### 2.3.1 Biogenesis

Mitochondrial biogenesis is the process through which pre-existing mitochondria grow and divide. It contributes to maintain cellular metabolic homeostasis by providing a pool of healthy mitochondria and eliminating damaged mitochondria. Mitochondrial biogenesis requires proteins encoded by both mitochondrial and nuclear genomes. It involves peroxisome proliferator-activated receptor gamma (PPARγ) coactivator1 α (PGC1α), a co-transcriptional factor which drives biogenesis especially by activating nuclear respiratory factor (NRF) 1 and 2. NRF1 and NRF2 are both transcriptional factors which activate the mitochondrial transcription factor A (TFAM), which in turn drives transcription and replication of mtDNA. NRF1 and NRF2 also control the expression of nuclear genes encoding ETC subunits and proteins required for mitochondrial functions [21]. Mitochondrial biogenesis can be influenced by cellular factors, among which oxidative stress and proliferation or differentiation state [22].

#### 2.3.2 Fusion and fission

Mitochondria are submitted to processes of fusion and fission. The fission is the phenomenon by which a mitochondrion divides into smaller daughter mitochondria. During cell division, it thereby generates the necessary number of organelles for redistribution into daughter cells. Fission can also occur to facilitate the autophagy of depolarized mitochondria, characterized by a low mitochondrial membrane potential and impaired ATP production [23]. Fusion allows the cell to generate the correct number of mitochondria for growing cells on one hand while on the other hand, it is a process by which each mitochondrion compensates defects of each other [24]. Fusion machinery indeed allows mtDNA, protein and membrane component exchanges between mitochondria which thus provide essential support to ensure mitochondrial function [25]. While a balance between fission and fusion maintains a stable mitochondria number within a cell by regulating mitochondrial morphology and bioenergetic functionality, changes in mitochondrial fusion or fission balance can occur as a cellular response to stress. An imbalance of fusion over fission results in elongated and tubular mitochondria, constituting a mitochondrial tubular network, whereas an imbalance towards fission generates fragmented mitochondria [26], often associated with decreased ATP production and considered as pathological [27,28]. While alterations in mitochondrial morphology is associated with impairment of mitochondrial energy production [[29], [30], [31], [32]], mitochondrial metabolism in turn controls mitochondrial morphology. In case of metabolic stress, mitochondria tend to fuse in order to increase their oxidative capacity thus maximizing OXPHOS and energy production [33]. Mitochondrial function is thus strongly connected to mitochondria morphology as changes in mitochondria morphology (e.g. shape, cristae integrity) impact mitochondria bioenergetic state and *vice versa* [34].

#### 2.3.3 Mitochondrial quality control

##### 2.3.3.1 Mitochondrial unfolded protein response

One mechanism of mitochondrial quality control is the mitochondrial unfolded protein response (mtUPR). This pathway is triggered by the accumulation of misfolded proteins in mitochondria caused, for example, by excessive reactive oxygen species (ROS) production [35]. mtUPR is thus able to reduce mitochondrial proteotoxic stress and reestablish protein homeostasis by increasing the mitochondrial chaperone and protease pool through their transcription by the nuclear activation of the transcription factor C/EBP homologous protein (CHOP) [35]. Mitochondrial proteotoxic stress also induces the expression of NRF1, involved in mitochondrial biogenesis [35]. mtUPR also displays regulatory axis, based on the sirtuin (SIRT) 7 and NRF1 interplay, in which mitochondrial protein folding stress, in response to nutrient deprivation, induces SIRT7 expression which in turn repressed NRF1 and leads to diminished mitochondrial activity and biogenesis to avoid cell death [36]. This mechanism thus promotes cell quiescence and nutritional stress resistance [36].

mtUPR also displays a mitochondrial stress resistance machinery that involves the mitochondrial deacetylase SIRT3 in a CHOP-independent manner [37]. SIRT3 axis of mtUPR has indeed been shown to limit oxidative damages in stressed mitochondria by inducing the expression of the antioxidant enzyme superoxide dismutase (SOD) 2, through the activation of the nuclear transcription factor FOXO3, and potentiates the elimination of irreparable mitochondria by activating mitophagy targets [35].

mtUPR thus regulates the activation of genes involved in metabolic pathways, ROS scavenging machinery and mitochondrial dynamics, that sustains the reestablishment of protein homeostasis as well as metabolism to withstand mitochondrial stress and maintain mitochondrial network integrity [38].

##### 2.3.3.2 Mitophagy

Autophagy is a catabolic pathway which removes cytoplasmic components, including damaged organelles, by lysosomal degradation. Applied to mitochondria, this process is called mitophagy (Figure 1) and results in the elimination of damaged mitochondria, marked by depolarized IMM or excess of unfolded proteins beyond the mtUPR repair limit, to prevent excessive mitochondrial ROS (mtROS) generation and cell toxicity [39]. Mitophagy seems to be also connected to mitochondrial motility. Wang et al. suggested that, during mitophagy, combination of PINK1 and Parkin activities contributes to isolate the damaged mitochondria from the kinesin network by the proteasomal degradation of Miro [40] as well as down-regulation of fusion mediators, decreasing probability of mitochondria to fuse with others [41].

### 2.4 Mitochondrial bioenergetics

Mitochondria is considered as the powerhouse of the cell which provides energy in the form of ATP. This energy is produced through OXPHOS which consists in oxidation of the redox cofactors nicotinamide adenine dinucleotide (NADH) and flavin adenine dinucleotide (FADH_2_) coupled with the phosphorylation of ADP into ATP.

**Figure 2:**
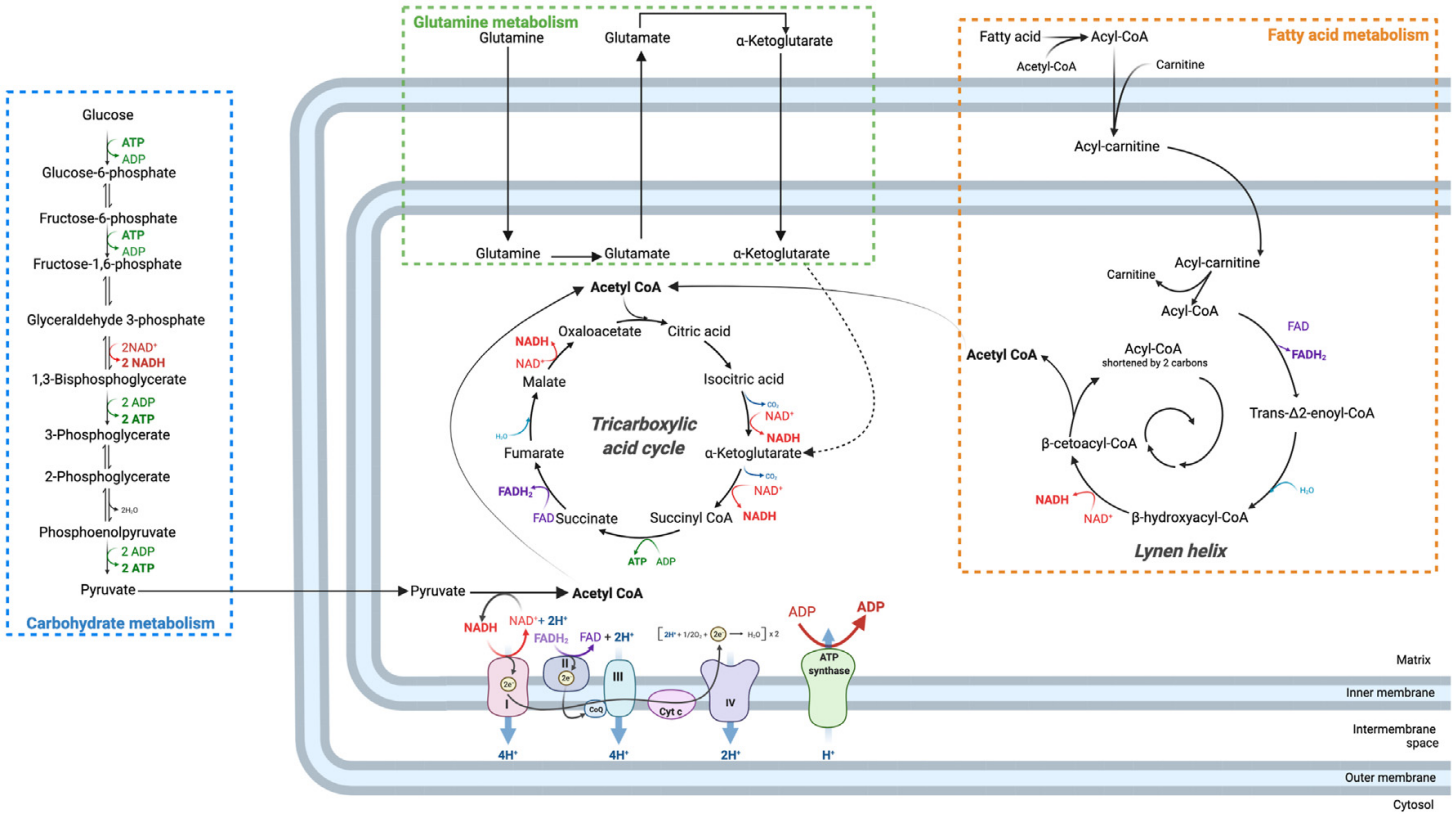
**Schematic view of mitochondrial bioenergetics in intestinal epithelial cells.** Electron transport chain (ETC) is fueled through catabolism of Acetyl-CoA in the tricarboxylic acid cycle (TCA) that generates NADH and FADH_2_, whose oxidation in complexes I and II of the ETC initiates electron transfer between complexes. Acetyl-CoA is a metabolic crossroad since it can be obtained through carbohydrate catabolism as well as fatty acid β-oxidation in the Lynen helix, including butyrate and fatty acids from diet, that also generates NADH and FADH_2_ in each round. Amino acid catabolism also fuels TCA notably through conversion of glutamine into glutamate and then dehydrogenation into α-ketoglutarate.

NADH and FADH_2_ is obtained through catabolic pathways such as fatty acid β-oxidation via the Lynen helix or carbohydrate catabolism, which includes glycolysis, or through amino acid catabolism (Figure 2). Those pathways give rise to acetyl-CoA that enters in the TCA, also known as Krebs cycle, in which each oxidative decarboxylation generates NADH. Acetyl-CoA is thus considered as a metabolic crossroad.

**Figure 3:**
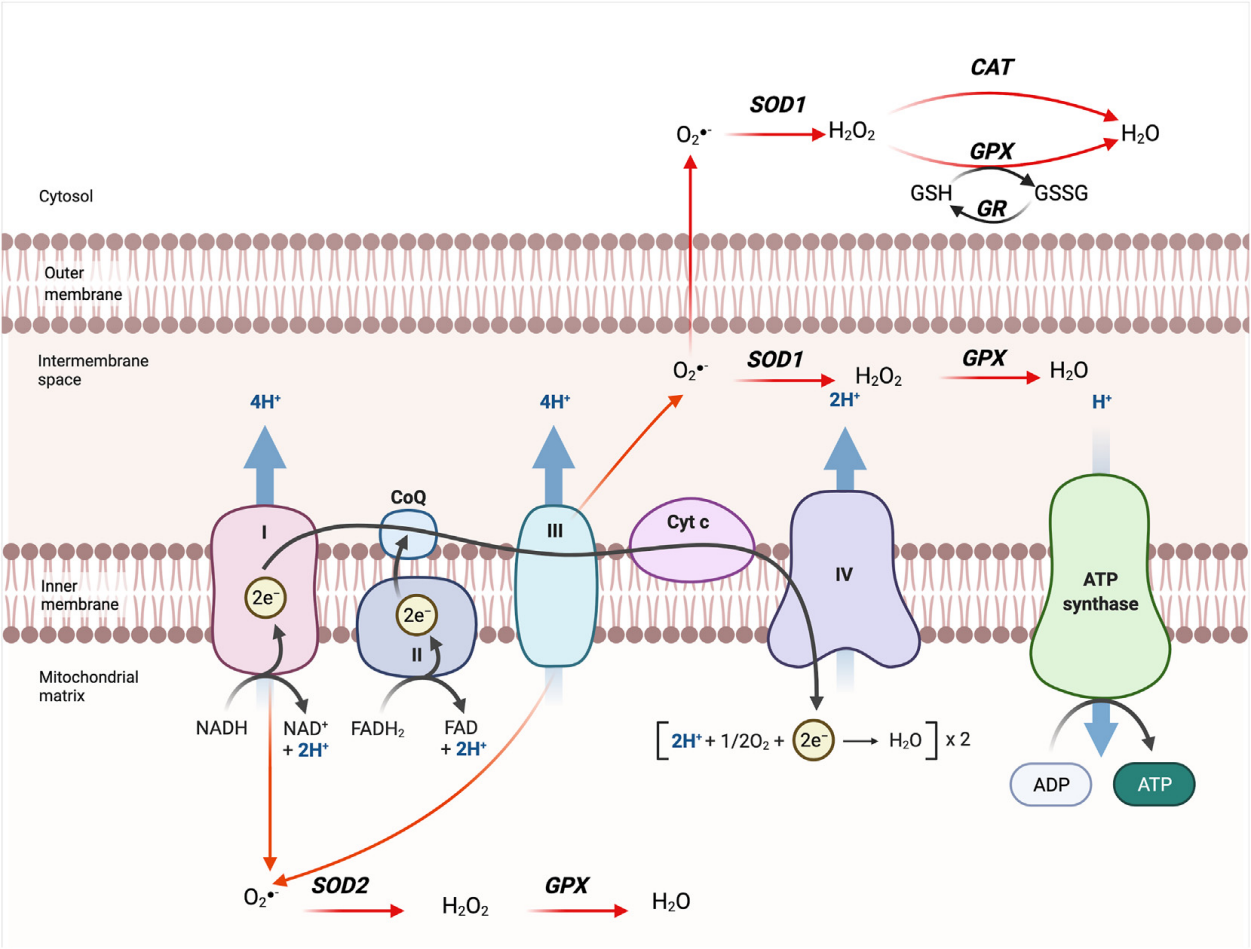
**Electron transport chain (ETC) and Reactive Oxygen Species (ROS) generation.** ETC is made of 5 enzymatic complexes: **NADH dehydrogenase** (Complex I), **succinate dehydrogenase** (Complex II), **ubiquinol cytochrome c oxidoreductase** (Complex III), **cytochrome c oxidase** (Complex IV) and **ATP synthase** (Complex V). By respectively binding to complex I and II, NADH and FADH_2_ provides each two electrons to the ETC during their oxidation. These electrons are transferred, via several iron sulfur clusters, to lipid soluble redox coenzymes Q (CoQ). Then, coenzyme Q reduction transfers electrons to Complex III which are in turn transferred to cytochrome c (Cyt c) molecules. Cytochromes c next provide electrons to Complex IV that catalyzes the reduction of 2 O_2_ molecules into H_2_O. In each complex, except Complex II, energy of passing electrons is accompanied by proton pumping from matrix to the intermembrane space (IMS). Accumulation of protons in the IMS generates a potential difference (electrical and chemical gradients) across the inner membrane. Those gradients in turn drive the transport of protons from IMS to the matrix through complex V, also known as ATP synthase, and constitutes a proton-driving force that drives the rotation of Complex V subunits. This mechanical energy finally allows chemical synthesis of ATP from condensation of inorganic phosphate and ADP. Electron leaks from complexes I, toward mitochondrial matrix, and III, into the matrix and into the IMS, interact with O_2_ and generate superoxide anions. The latter may then be converted into H_2_O_2_ and H_2_O by antioxidant enzymes (SOD1/2: superoxide dismutase 1/2; GPX: glutathione peroxidase; GR: glutathione reductase; GSH: reduced glutathione; GSSG: oxidised glutathione; CAT: catalase) in the cytosol or within IMM or mitochondrial matrix.

During their oxidation, NADH and FADH_2_ transfer their electrons, initiating the electron flux through the ETC, also known as respiratory chain (Figure 3). This chain is made of 5 enzymatic complexes: NADH dehydrogenase (Complex I), succinate dehydrogenase (Complex II), ubiquinol cytochrome c oxidoreductase (Complex III), cytochrome c oxidase (Complex IV) and ATP synthase (Complex V) [42]. Electron transfer through ETC complexes is accompanied with proton pumping, from mitochondrial matrix toward IMS that generates electrical and chemical gradients. Those gradients in turn drive the transport of protons from IMS to the matrix through ATP synthase and constitute a proton driving force that drives the rotation of ATP synthase subunits. This mechanical energy finally allows chemical synthesis of ATP from condensation of inorganic phosphate and ADP.

### 2.5 Mitochondrial ROS generation

Mitochondria are the most abundant source of ROS. ROS include superoxide radicals (O_2_^⦁-^) and downstream products such as hydroxyl and peroxide compounds, like hydrogen peroxide (H_2_O_2_). OXPHOS is indeed not totally efficient as 0.2–2% of the electrons leak out, notably from complexes I and III, and interact with O_2_ to produce ROS, especially O_2_^⦁-^ [43] (Figure 3). Some mitochondrial enzymes also constitute, to a lesser extent, a source of mtROS. First, the matrix-located pyruvate and α-ketoglutarate dehydrogenases are reduced flavoproteins that constitute a source of O_2_^⦁-^ and H_2_O_2_ through electron transfer by their flavin subunit [44]. Then, mitochondrial fatty acid β-oxidation is also a source of ROS whether by favoring electron leaks from the ETC via complexes I and III and electron transfer flavoprotein and oxidoreductase [45] or from enzymes involved in the mitochondrial β-oxidation such as the flavoprotein long-chain acyl-CoA dehydrogenase [46]. To a lesser extent, ROS are also produced in other cell compartments notably through cytosolic oxidases, such as xanthine oxidase [47], within the peroxisomes via their oxidases [48], through ER cytochrome P450 [49] and also from membrane enzymes, such as the family of NADPH oxidases Nox and Duox [50].

To balance ROS concentration, cells possess an endogenous antioxidant enzymatic machinery (Figure 3). Exogenous antioxidants, such as dietary vitamins and minerals, also exert a role in ROS balance [51]. However, an excess of ROS generation leads to cellular damages, via lipid peroxidation and protein oxidation as well as DNA and mtDNA damages, that ultimately triggers programmed cell death [52]. mtDNA, because of its structure, is more susceptible to ROS damages, resulting in single or double mtDNA breaks or DNA base alterations such as bypasses of the thymine and formation of thymine glycol [53]. Moreover, TCA enzymes are also vulnerable to ROS injuries [54,55] making mitochondria the primary target of ROS-induced cellular damages. It is noteworthy that ROS also display benefic roles in tissue homeostasis. As second messengers, they are involved in multiple intracellular transduction pathways and control the action of mitogen-activated protein kinases [56].

## 3 Mitochondrial functions in intestinal epithelial cells

### 3.1 Epithelial renewal dynamics in small intestine and colon

The small intestine epithelium is characterized by elongated domains towards lumen, called villi, composed of differentiated IEC and inward-invaginated domains called crypts, which contain ISC. Colonic epithelium only displays crypts and a flat epithelial surface.

The intestinal epithelium is in constant turn-over and self-renews every 4–5 days. Crypt-based columnar ISC are located at the bottom of the crypts, intercalated between Paneth cells in the small intestine, and harbor leucine-rich repeat containing G protein-coupled receptor 5 (Lgr5) gene as a specific marker [57]. Here, they continuously undergo asymmetric division generating transit-amplifying cells. Through continuous and rapid cycles of proliferation, these progenitor cells gradually move upward the crypt and finally differentiate into absorptive IEC (enterocytes or colonocytes) or secretory cells (such as goblet or enteroendocrine cells). Paneth cells remain within the small intestinal crypts where they pursue their role of antimicrobial defense and stem cell maintenance since they participate to the niche environment of Lgr5+ cells. An equivalent to Paneth cells in the colon is Reg4+ cells which also play a role in epithelial niche for ISC [58]. Finally, at the top of the villus in the small intestine, or at the colonic epithelial surface, senescent cells lose their attachment to the basement membrane and neighboring cells, and fall into the lumen via anoikis [59].

Because intestinal epithelium is exposed to pathogens and luminal components derived from diet or microbiota, which may trigger cell damages and apoptosis, epithelial renewal is essential to ensure intestinal homeostasis through intestinal barrier integrity [60]. Yet, this process requires high amount of energy directly provided by mitochondria.

### 3.2 Intestinal epithelium zonation associated with different metabolic activity along the crypt/villus axis

**Figure 4:**
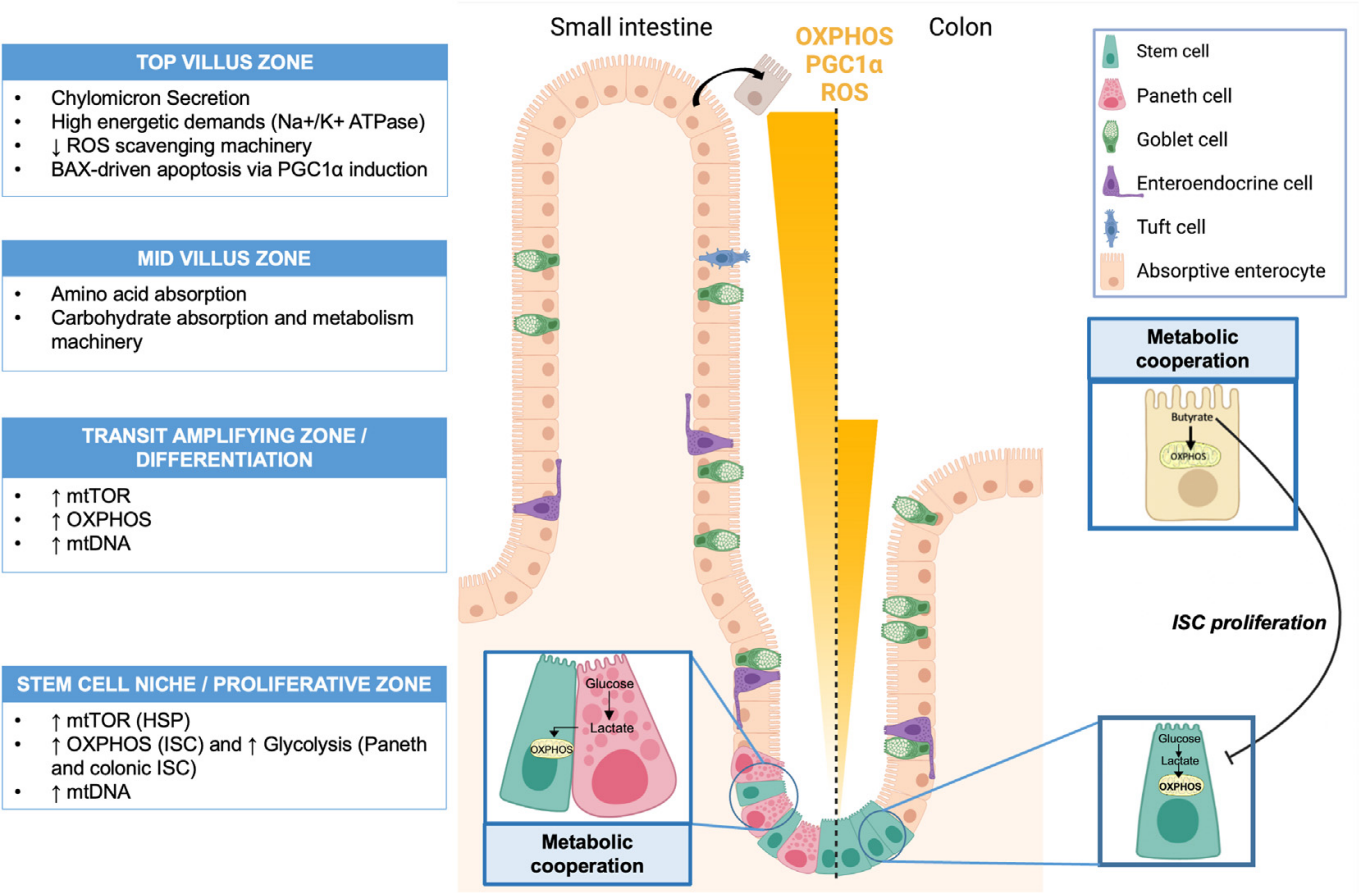
**Intestinal epithelium zonation and metabolic activities along the crypt/villus axis**. As intestinal epithelial cells (IEC) migrate up along the crypt/villus axis, they display a metabolic gradient associated with different mitochondrial activities that play a major role in intestinal homeostasis. Intestinal crypts house intestinal stem cells (ISC) that rely on glycolysis to rapidly provide energy needed for proliferation, in association with oxidative phosphorylation (OXPHOS) in the small intestine, supported by Paneth cell-derived lactate. Then, IEC migration upward the crypt/villus axis is accompanied with higher mitochondrial content and PGC1α expression, notably induced by the mammalian target of rapamycin (mTOR), and higher OXPHOS activities to support essential functions such as nutrient absorption. Enterocytes from small intestine mainly rely on d-glucose, l-glutamine and l-glutamate to fuel OXPHOS while differentiated colonocytes mostly β-oxidize microbiota-derived butyrate thus protecting ISC against its anti-proliferative effect. At villi tips or at the top of colonic crypts, enhanced metabolic activities finally generate reactive oxygen species (ROS) accumulation in mature IEC which contributes to trigger apoptosis by anoikis.

As they migrate upward the crypt-villus axis, enterocytes encounter zonated cell states associated with different functions, depending on the extra-cellular environment and gene expression, and thus different metabolic activities. By tracing Lgr5+ ISC progenies, it appears that most genes are not continuously expressed along the crypt-villus axis, suggesting that enterocytes are not terminally differentiated during their migration along the villi, but rather continuously transdifferentiate [61]. Heatmaps and zonation profiles of genes involved in the absorption of distinct nutrient classes highlight a zonation of nutrient absorption machineries along the crypt-villus axis in mouse jejunum. Mid-villus enterocytes display amino acid and carbohydrate absorption and metabolism machinery, whereas villus tip cells have increased expressions of genes involved in lipid absorption and chylomicron secretion [61] (Figure 4).

In connection with the intestinal epithelium zonation, IEC display a metabolic gradient associated with their proliferation and phenotypic transitions during their migration along the crypt-villus axis. Paneth cells tend to have a high glycolytic phenotype whereas Lgr5+ ISC display both high glycolytic and OXPHOS activities as shown by NADH/NAD + fluorescence analyzes in mouse small intestine through three-dimensional phasor fluorescence lifetime microscopy [62]. On the contrary, differentiated enterocytes at the top of the villi [63] or colonocytes at the top of the crypts [64] use OXPHOS to fulfill their energetic needs (Figure 4). In line with this metabolic gradient, mitochondrial biogenesis and activity are also regulated along the crypt-villus axis. In rat small intestine IEC from villi contain at least twice more mitochondria than IEC from crypts. Those mitochondria also seem larger in villi than in crypts [65]. Moreover, in intestinal organoids, IEC differentiation has been linked to an increase in mitochondrial number [62].

Finally, ROS can regulate cell phenotypic shift along the crypt-villus axis. Immunohistochemistry analysis of mouse small intestine reveals that p38, a MAP kinase sensitive to redox status and involved in IEC differentiation, is activated at the bottom of intestinal crypts whereas it is less active in differentiated villi. In mouse small intestine organoids, p38 is activated during crypt formation and differentiation in responses to ROS signaling [66]. Furthermore, impairing OXPHOS in mouse small intestine organoids, by blocking ETC complexes or treating them with antioxidants, altered crypt formation [61]. Taken together those results suggest that mitochondrial function, through ROS signaling and p38 activity, drives crypt formation and IEC differentiation.

### 3.3 Mitochondrial function in the control of ISC homeostasis

Strong evidences suggest that cell metabolism is a key regulator of pluripotency and differentiation: metabolism could control stem cell fate and even determine cellular phenotype [67]. Generally, cells in active proliferation state perform high glycolytic activity and show low OXPHOS level [68], assimilable to the “Warburg effect”, a metabolic alteration of cancer cells characterized by important glycolysis, followed by lactic acid fermentation [69]. As highly proliferative cells, Lgr5+ cells exhibit high glycolytic activity. However, they also display relatively high mitochondrial activity and use lactate to produce ATP via sustained OXHPOS as observed in mouse small intestine organoids [62]. Indeed, besides supporting ISC stemness by providing essential growth factor, Paneth cells also support Lgr5+ cell metabolic state by providing glycolysis-derived lactate. Thus, in the small intestine, cells show different but cooperative metabolic activities (Figure 4). Yet, recent works demonstrated that fatty acid β-oxidation play a major role in ISC maintenance and stemness [70,71] mediated by the transcription factors HNF4A and HNF4G [72], both regulators of intestinal fatty acid oxidation. Furthermore, PRDM16, a transcription factor that promotes fatty acid β-oxidation through PPAR activation, is necessary for the survival of the stem-cell-derived progenitor cells in upper parts of the small intestine [73].

Paneth cells being absent in the colon, colonic ISC perform themselves the conversion of glucose into lactate through anaerobic glycolysis, even in presence of O_2_, thus harboring a Warburg-like metabolism [68]. Moreover, a metabolic barrier is established in mammalian colonic crypts, also called “butyrate paradox”, that protect ISC from the anti-proliferative effect of butyrate. This short-chain fatty acid (SCFA) produced by the gut microbiota, can indeed inhibit ISC proliferation at physiological concentrations through the inhibition of histone deacetylase (HDAC), and increasing FOXO3 binding on negative regulators of cell cycle genes and inhibiting proliferation of ISC [74]. Theoretically, since colonic ISC display higher rates of glycolytic activity than OXPHOS [68], butyrate would not be efficiently oxidized and would accumulate in the cytoplasm and inhibit consequently proliferation [74]. However, colonocytes at the top of the crypt preferentially oxidize butyrate thus limiting its amount at crypts basis, where ISC are located, and favoring ISC proliferation [74] (Figure 4).

Furthermore, mitochondria are able to modulate ISC stemness and proliferation via mtUPR and notably through mitochondrial HSP [75]. To assess its role in intestinal homeostasis, the mitochondrial chaperone HSP60, required for the folding and assembly of proteins imported into mitochondria [76], was deleted in mouse intestinal epithelium. This resulted in mtUPR activation and mitochondrial function impairment in association with a loss of stemness and cell proliferation in Hsp60-deficient jejunal crypts [75], suggesting a critical role for mitochondrial function and mtUPR in maintaining stemness and proliferation.

### 3.4 Influence of intracellular signaling on mitochondrial bioenergetics zonation

As mitochondrial biogenesis modulator, PGC1α can act as a metabolic regulator of IEC fate by increasing OXPHOS and fatty acid β-oxidation [77]. PGC1α is predominantly expressed at the top of the villi (and far less in proliferative crypts) along with PPAR-γ [78], with which it interacts to trigger gene expression. As an epithelial nuclear receptor primarily synthesized in differentiated colonocytes and activated by butyrate, PPARγ triggers mitochondrial β-oxidation of fatty acids and oxygen consumption through OXPHOS, further contributing to the high OXPHOS activity at the top of crypts [64,79]. PPARγ and PGC1α thus seem to contribute to the intestinal metabolic gradient along the crypt-villus and crypt axes.

Moreover, along the crypt-villus axis, the mammalian target of rapamycin (mTOR) increases levels of mtDNA and the expression of genes involved in OXPHOS in IEC, through nutrient signaling, such as amino acids and glucose, and growth factors, such as insulin growth factor [80,81]. Also, the transcriptional repressor protein Yin Yang 1 (YY1) and mTOR play critical roles in intestinal epithelium repair and ISC renewal since their deletion in mice impairs stem cell renewal [82,83]. In muscle stem cells, mTOR has been shown to mediate the complex formation between PGC1α and YY1 resulting in increased mitochondrial biogenesis and OXPHOS gene expression [84]. Whether this also applies in IEC need further investigations.

### 3.5 Mitochondrial function and apoptosis through PGC1α-driven ROS accumulation

The overexpression of PGC1α and enhanced OXPHOS in differentiated IEC is also accompanied by increased mtROS production. However, in the intestine, contrarily to other tissues where it induces the expression of antioxidant enzymes [85], PGC1α overexpression enhances mitochondrial activity and ROS accumulation, without displaying any effect on ROS scavenging [86]. Furthermore, accumulation of intracellular free radicals decreases antioxidant mitochondrial enzyme activities (SOD, GPX and CAT) in an *in vitro* model of pig enterocytes [87]. This raises the question of mitochondrial injury consequences on intestinal homeostasis.

While PGC1α does not display antioxidant properties in the small intestine, PGC1β, a PGC1α homolog, localized all along the entire crypt-villus axis in the small intestine and crypts in the colon, triggers OXPHOS but also protects the intestinal epithelium against ROS-driven damages by stimulating antioxidant enzymes production, such as SOD2 and GPX4 [88]. Indeed, overexpressing *Pgc1β* in mouse resulted in higher enterocyte lifespan and increased tumor susceptibility while *Pgc1β* knockout mice were protected against intestinal carcinogenesis [88]. Hence, this transcriptional factor coactivator orchestrates the balance between enhanced mitochondrial activity and protection against ROS over-production along the entire epithelium in the small intestine and colon. Yet, enzymes of the antioxidant machinery, such as SOD2 and CAT, are poorly expressed in villi tips [89] compared to crypt base, while prooxidant enzymes, such as glutathione transferase/reductase, are highly expressed in it [89]. Thus ROS accumulation ultimately fosters cell apoptosis at the top of the crypt-villus axis [77].

### 3.6 Energetic substrates of IEC

IEC are polarized cells facing the intestinal lumen on one side and the *milieu interieur* on the other side. While the *milieu interieur* composition is relatively stable in healthy individuals thanks to strong homeostasis regulatory mechanisms, the composition of the luminal side can change drastically depending on the diet, time of the day and gut microbiota composition and activities. Indeed, the composition of ingested meals as well as diet patterns directly modify the luminal composition of the small intestine. Moreover, and although the growth of microorganisms is globally inhibited by bile acids and pancreatic secretions in the small intestine [90], the role of jejunal bacteria on nutrient absorption, and especially lipid absorption, is more and more recognized [91,92]. As for the colon, it constitutes an important place of bacterial fermentation. Hence, in the colon, undigested or partially digested dietary carbohydrates [structural polysaccharides of plant cell walls, oligosaccharides (galactooligosaccharides or fructooligosaccharides [93]), resistant starch [94]], dietary proteins and lipids or substrates from endogenous sources (digestive secretions, exfoliated cells, and mucins) are metabolized by gut bacteria, resulting in variable luminal composition, depending on dietary factors and/or microbiota composition [95].

This particularity of the IEC environment leads to a large possibility of energetic substrates for those cells (Figure 2). In general, enterocytes mostly use d-glucose, l-glutamine and l-glutamate as substrates for oxidative metabolism [96,97], and colonocytes produce energy from substrates originating either from the lumen, such as SCFA, mainly butyrate, or to a lesser extent from blood origin, such as d-glucose, acetoacetate and l-glutamine [98]. However, many substrates and metabolites, directly originating from the diet or from gut microbial transformation, are also used as energetic fuels and modulate IEC mitochondrial function (Table 1).

**Table 1:** Effects of dietary and gut microbial-derived metabolites on mitochondrial function of intestinal epithelial cells.

Metabolite	Luminal concentration	Origin	Target tissue	Fate^a^ and effect on IEC mitochondria
Glucose	300 mM in intestinal chyme [147]	Diet	Small intestine (from lumen)/Colon (from bloodstream)	• Aerobic metabolism: conversion into pyruvate in the cytosol and OXPHOS of pyruvate [148] • Anaerobic glycolysis in presence of limited amount of oxygen [148]
l-glutamine and l-glutamate	l-glutamate found at 0.1 mM before meal to 2.6 mM (3 h after meal containing 50 g of purified bovine serum albumin) [149]	Diet	Small intestine (from lumen)/Colon (from bloodstream)	• l-glutamine: first converted into l-glutamate and ammonia via the mitochondrial phosphate-dependent glutaminase [150], then cytosolic transamination into l-glutamate. • l-glutamate: hydrolysis via peptidase to release glutamate, then transamination with oxaloacetate to form α-ketoglutarate and l-aspartate, both oxidized by TCA cycle and OXPHOS [12,150]
Indole	Up to 1 mM in human feces [151]	Produced by gut microbiota from tryptophan [151]	Colon	• At 1 mM *in vitro* on GLUTag cells: blocking of mitochondrial complex I activity of entero-endocrine cells thus impairing OXPHOS [152] • At 2.5 mM *in vitro* on HT-29 Glc−/+: decreases mitochondrial oxygen consumption and maximal respiration, provokes oxidative stress [153]
H_2_S	In the large intestinal lumen, H_2_S is present at concentrations ranging from 1.0 to 2.4 mM [154] and human fecal sulfide concentrations can reach 3.4 mM in association with high-meat diet [155]	Bacterial metabolite mainly produced through cysteine catabolism by gut bacteria [156]	Colon	• Detoxification through oxidation by the mitochondrial sulfide oxidized unit in HT-29 Glc −/+ and in human colonic biopsies [157] • Under 20 μM *in vitro* on HT-29 Glc −/+ cells, ATP production through H_2_S oxidation [157] • At concentrations ranging from 37.5 μM to 62.5 μM *in vitro* on HT-29 cells, decreases colonocytes oxygen consumption in a reversible way via reduction of cytochrome c oxidase activity and increase in proton leak thus impairing ATP production and promotes glycolysis [158] • At 0.1 mM *in vitro* on HT-29 and HCEC cells: limited renewal of oxidized coenzymes, essential for ETC activity, through the decrease in NAD+/NADH ratio [159] • At millimolar concentrations *in vitro* on HT-29: inhibitions of l-glutamine and butyrate mitochondrial oxidation as a consequence of the H_2_S-driven inhibition of the cytochrome c oxidase activity [158]
P-cresol	Around 0.4 mM in human feces [160]	Produced from l-tyrosine by gut microbiota [160]	Colon	• At concentrations above 0.8 mM *in vitro* on HT-29 Glc −/+: mitochondrial dysfunction by increasing proton leak through IMM, causing impairment of ATP production and concomitant production of anion superoxide [161] • At 1.6 mM on rat normal colonocytes: inhibits oxygen consumption [161]
4-hydroxyphenylacetic acid (HO-PAA)	Micromolar concentrations [162]	Produced from l-tyrosine by gut microbiota [162]	Colon	At 1 mM *in vitro* on HT-29 Glc−/+:: decreased mitochondrial complex I activity and colonocytes mitochondrial respiration, increased ROS levels [163]
Ammonia (NH_4_^+^ and NH_3_^+^)	Up to 30 mM in human feces [164]	Deamination of amino acids and urea hydrolysis by bacteria [165]	Colon	• At millimolar concentrations in rat colonocytes: decrease in basal oxygen consumption [166] and inhibition of SCFA β-oxidation, possibly by blocking the TCA enzyme malate dehydrogenase in case of low glucose availability [167] • At 60 mM *in vitro* on Caco-2: decreased expressions of genes encoding mitochondrial ETC subunits, reduced cellular ATP level, mitochondrial membrane potential and TCA intermediate contents associated with the emergence of oxidative stress and epithelial barrier disruption [168]
Butyrate	From 11 mM to 25 mM per kg of intestinal content [169]	Anaerobic bacterial fermentation of undigested carbohydrates obtained from dietary fibers but also of several amino acids, derived from undigested proteins or endogenous sources [170]	Colon	• Preferred energetic fuel of colonocytes, contributing to 70% of their energy requirements, through mitochondrial β-oxidation and TCA cycle [79,171] • Inhibition of other substrates oxidation, such as acetoacetate, l-glutamine and d-glucose in rat isolated colonocytes [98] • Regulation of IEC metabolism notably through PPAR-γ and Angiopoietin-like protein 4 induction *in vitro* on T84 and HT-29 cell lines and in mouse colonocytes [172]
Polyamines: putrescine, spermidine, spermine	Colonic putrescine and spermidine found at millimolar concentrations [173]	Diet and gut microbiota Produced from decarboxylation of l-ornithine, via ornithine decarboxylase (ODC1) activity, to form putrescine, the precursor of spermidine and spermine [174]	Small intestine for dietary polyamines [175], colon for gut-derived polyamines [173]	• In IEC from small intestine, OXPHOS substrate through conversion into succinate as shown in rat [174] • In mouse colonocyte, increased levels of ETC proteins in mouse colonocytes [176]

Abbreviation: ETC: electron transport chain, IEC: intestinal epithelial cells, IMM: inner mitochondrial membrane, OXPHOS: oxidative phosphorylation ROS: reactive oxygen species, SCFA: ShortChain Fatty Acids, TCA: tricarboxylic acid cycle.

a Except for the entero-endocrine cell line GLUTag, all cited cell lines are absorptive IEC.

## 4 Role of mitochondrial dysfunction in diet-induced obesity intestinal homeostasis disturbances

### 4.1 Impact of HFD on IEC mitochondrial function

#### 4.1.1 In the small intestine

##### 4.1.1.1 Metabolic switch towards lipid catabolism

Depending on the diet composition, the small intestine is “metabolically flexible” [99]. In response to excessive fat consumption, it increases fat absorption, esterification into triacylglycerol and export into chylomicrons [100], even after a short-term exposure of 3 days in humans [101]. Triacylglycerol that have not been exported are stored in cytosolic lipid droplets (CLD) within the enterocytes, up to 18 h in humans [102,103] and 12 h in mice [104], and can be remobilized later, notably after glucose ingestion [105,106]. Functional analysis and network annotation performed on CLD fractions from obese (60% kcal from fat for 12 weeks) and lean (10% kcal from fat) mice subjected to oil gavage, revealed that lipid catabolism is the second most enriched gene ontology biological process (GO_BP) and is more present in the proximal parts of the small intestine in both obese and lean mice after oral gavage [107]. Those enriched GO_BP include proteins involved in mitochondrial β-oxidation (Acyl-CoA Dehydrogenase Very Long Chain, Acetyl-CoA Acyltransferase 2, Hydroxyacyl-CoA Dehydrogenase Trifunctional Multienzyme Complex Subunit Alpha and Electron Transfer Flavoprotein Subunit Beta) and are overexpressed in CLD fractions of enterocytes from obese mice [107,108].

In response to increase dietary fat absorption, the small intestine is marked by metabolic adaptations towards fatty acid β-oxidation [[109], [110], [111]]. Interestingly, three days of HFD consumption (60% kcal from fat) is sufficient to decrease nearly by half the gene expression of the glucose transporter Slc5a1 and of hexokinase 1 (Hk1) in jejunal isolated enterocytes [111], suggesting a decrease in glucose metabolism in enterocytes of HFD mice. On the other hand, protein expression of HMGCS2, a mitochondrial enzyme involved in ketogenesis, is highly increased in the jejunum of HFD-fed mice. Ketogenesis is performed from acetyl-CoA generated via fatty acid β-oxidation to produce energy from lipids, in a context of TCA intermediate deprivation. This suggests a metabolic re-programming of jejunal enterocytes from glycolysis towards lipid metabolism, from 3 days after HFD consumption [111] to 20 weeks of HFD [110]. To predict metabolic flux variations in response to increased dietary lipid intakes, a constraint-based metabolic model of mitochondria from murine enterocyte was developed [112]. Simulations and protein transcript analysis predicted an increase in β-oxidation in response to increased ratio of lipid/carbohydrate in the diet. Kondo et al. besides showed that 2 weeks of HFD consumption in C57BL/6 J mice increased carnitine palmitoyltransferase (CPT) activity, that allows fatty acid entrance within mitochondria, and the levels of [U–^14^C]palmitic acid β-oxidation in mitochondrial fractions of jejunal epithelial cells from HFD-fed mice [109].

Despite the fact that fatty acids are sparsely used as energetic substrate by enterocytes in physiological states [97], increased β-oxidation can be seen as a mechanism to counteract excessive lipid storage as found in hepatocytes in a context of nonalcoholic fatty liver disease [113].

##### 4.1.1.2 Role of IEC lipid metabolism in the onset of obesity

The ability to catabolize dietary fatty acids in enterocytes seems directly associated with protection against obesity development. By comparing levels of expression of fatty acid metabolism-related genes in response to a HFD between C57BL/6 J mice and an obesity-resistant strain (A/J), it appears that expressions were higher in HFD-fed A/J mice while CPT activity displayed increased expression in A/J mice compared to the HFD-fed C57BL/6 J mice [109]. Moreover, intestine-specific deletion of HDAC3 protects mice fed a HFD from obesity [114]. HDAC3 deletion increased fatty acid β-oxidation in IEC from duodenum compared to wild-type mice. After 17 weeks on HFD, mice lacking intestinal HDAC3 had less triglyceride storage in their enterocytes than control mice in HFD and similar bodyweight than mice fed a standard diet [114]. Likewise, hypothesizing that enterocyte lipid metabolism plays a major role in controlling obesity development and metabolic alterations, Ramachandran et al. developed a mutant mouse model which overexpresses the mitochondrial transporter CPT1a in their enterocytes (iCPT1mt). iCPT1mt mice fed a HFD exhibited increased levels of β-oxidation in IEC compared to floxed mice (Cpt1mt^fl/fl^). Although iCPT1mt HFD mice developed obesity, with similar body weight gain than control mice, their visceral fat mass was reduced, and they displayed improved glycemic control compared HFD Cpt1mt^fl/fl^ [115]. Moreover, overexpression of SIRT3 in enterocytes increased metabolic activities in jejunal enterocytes after oral gavage with oleic acid in HFD mice, with notably lower concentrations of palmitoyl-CoA, indicating increased β-oxidation [116]. Furthermore, SIRT3 overexpression in IEC improves mouse glucose homeostasis and protects against insulin resistance under HFD [116]. Besides, SIRT3, in association with SIRT5, is also able to enhance fatty acid β-oxidation by promoting very long chain acyl-CoA dehydrogenase activity and its binding to cardiolipin of mitochondrial membranes [117].

Taken together, these data indicate that enhancing fatty acid β-oxidation in the jejunum of HFD mice improves body glucose homeostasis, prevents insulin resistance, and reduces fat mass gain.

##### 4.1.1.3 Lipid catabolism and risks of oxidative stress

Despite the fact that enhanced fatty acid β-oxidation improves glucose homeostasis and reduces adiposity, it could however induce oxidative stress [118,119]. Fatty acid β-oxidation indeed constitutes a source of mtROS as described in *2.5*. Furthermore, HFD consumption has been linked to reduced concentrations of glutathione and antioxidant enzymes (SOD and CAT) in duodenal homogenates from rats fed a HFD (45% kcal fat) for 4 weeks. Additionally, increased ROS detection has been observed in duodenal homogenates of mice fed a HFD (21% kcal fat) for 8 weeks [118] and in the ileum of rats fed a high-fat/high-sucrose diet (23% kcal from fat) for 17 weeks [119]. Interestingly, genetically obese rats do not show any difference in ROS detection in the intestine compared to control mice suggesting that oxidative stress is related to high fat/high sucrose consumption rather than obesity itself in the small intestine [119].

##### 4.1.1.4 Mitochondria function from IEC of small intestine in HFD

Although several studies have pinpointed the metabolic switch toward fatty acid metabolism that occurs in IEC from the small intestine under HFD, no precise description of mitochondrial function within those enterocytes is available so far. In CLD fractions from obese (60% kcal from fat for 12 weeks) and lean (10% kcal from fat) mice subjected to oil gavage, the most enriched GO_BP terms include proteins involved in mitochondrial ATP synthesis [107]. Consistent with the fact that proteins involved in mitochondrial β-oxidation were mostly found in CLD fractions from enterocytes of obese mice, the authors suggested that enterocytes may adapt against excessive fatty acid absorption by associating CLD and mitochondria, thus favoring fatty acid catabolism to compensate their storage [107]. As for mitochondrial dynamics, the *in silico* model of mouse enterocyte exposed to increased ratio of lipid/carbohydrate in the diet suggested that mitochondrial fusion and fission were unaltered in enterocytes in a context of HFD consumption [112] although no *in vivo* nor *in vitro* analysis of mitochondrial dynamics has been yet performed to corroborate those predictions. Finally, 20 weeks of HFD consumption decreased by half the *Pgc1α* gene expression of jejunal enterocytes [115,116]. However, precise mechanisms that could explain the drop of *Pgc1α* have not been elucidated so far but could possibly be linked to HFD-induced oxidative stress, notably through enhanced lipid catabolism. Moreover, since *Pgc1α* is the master regulator of mitochondrial biogenesis, its decreased expression could affect mitochondrial number and/or enterocyte bioenergetics. However, altered mitochondrial biogenesis in response to HFD in intestine has not yet been demonstrated.

#### 4.1.2 In the colon

While the impact of HFD on IEC mitochondrial function in the jejunum is poorly described, several recent works show that HFD consumption is associated with alterations of mitochondrial function in colonocytes. The first indication was the observation of swollen mitochondria and decreased complexes II and III activities of freshly isolated mitochondria from colonic IEC of mice fed a HFD (60% kcal fat) for 16 weeks [120]. The time of HFD consumption or fat content of the diet does not seem to be crucial in this effect since shorter HFD consumption periods and lower fat content diets also resulted in signs of colonic dysfunction. Indeed, four weeks of a 45% kcal fat HFD induced a 50% decrease in epithelial ATP levels and lower expression of genes encoding for ETC subunits and *Sirt3* compared to colonocytes from control mice [121]. Likewise, HFD (60% kcal fat) consumption for 13 weeks resulted in decreased ATP levels in mouse colonic IEC and diminished expression of mRNA mitochondrial markers encoding for subunits V1 and S1 of mitochondrial complex I in these cells [122]. Furthermore, elevation of intracellular lactate levels in isolated colonocytes [121] and decreased pyruvate dehydrogenase activity, which catalyzes the conversion of pyruvate to Acetyl-CoA [121,122], suggest a switch of colonocyte metabolism from OXPHOS toward glycolysis.

To explain colonocyte mitochondrial dysfunction, the authors proposed mechanisms linked to dietary fatty acid metabolism. Although fatty acid β-oxidation, especially long chain and saturated fatty acids, elicits ROS production and mitochondrial bioenergetic impairments at the hepatic level [123,124], these alterations remain unclear in the intestine. *In vitro* models of IEC treated with palmitic acid to mimic HFD consumption all concluded on a negative effect of palmitic acid on mitochondrial function and an increase in ROS level (Table 2). However, fatty acid absorption primarily or even exclusively happens at the small intestinal level and not at the colonic level [125]. Thus, other mechanisms than increased dietary fatty acid metabolism have to be investigated to explain colonic mitochondrial dysfunction in HFD-fed mice. Among possible mechanisms, deleterious microbial metabolites have been proposed (see Table 1) as well as bile acids. Indeed, HFD consumption is correlated with elevated concentrations of bile acids in stools, which can reach a fecal concentration of 0.35 mM in humans fed a HFD [126]. In HT-29 colon cancer cells, deoxycholic acid increases mtROS generation, possibly by inhibiting complex III from mitochondria as reported in isolated rat liver mitochondria [127,128]. This excessive concentration of ROS generated by bile acid may potentiate mitochondrial damages. Furthermore, HFD consumption is associated with the emergence of sulphate reducing Desulfovibrionaceae in mice [129,130] and in obese humans [131]. Among Desulfovibrionaceae, *Bilophila wadsworthia* growth is mediated by the taurine-conjugated bile acid [129]. Taurine is indeed used by *B. wadsworthia* as a final electron acceptor of the ETC for anaerobic respiration which forms sulfide [132] and then, via dissimilatory sulfite reductase, hydrogen sulfide (H_2_S) [133]. Obese humans are also characterized by the growth of *Desulfovibrio piger* that degrades glycans from the host intestinal mucosa into H_2_S through sulfate reduction [131]. Considering the deleterious effects caused by high concentrations of H_2_S on mitochondrial function of IEC (Table 1), HFD may potentiate mitochondrial dysfunction by favoring the growth of sulfur-reducing bacteria and H_2_S formation.

**Table 2:** *In vitro* studies evaluating the effects of palmitate on mitochondrial function in different intestinal cell lines.

Concentration	Duration	Cell line	Results	Reference
100 μM	24 h	HCT-116	• Increased ROS production • Decreased mitochondrial membrane potential • Altered mitochondrial network • Treatment with n-acetyl-l-cysteine, a known ROS inhibitor, improved those defects	[134]
500 μM	24 h	NCI–H716	• Decreased maximal respiration and spare respiratory capacity • Alterations of mitochondrial membrane potential	[120]
From 1 mM to 2.5 mM	24 h	Caco-2	• Decreased ATP production rate from OXPHOS • Diminished expression of genes encoding subunits V1 and S1 of mitochondrial complex I (2.5 mM)	[122]

Abbreviation**:** OXPHOS: oxidative phosphorylation, ROS: reactive oxygen species.

In conclusion, several lines of evidence point to mitochondrial dysfunction in colonocytes while in-depth analysis of mitochondrial function in enterocytes has not been reported so far.

### 4.2 Possible link between HFD-induced mitochondrial dysfunction and alterations of epithelial homeostasis

#### 4.2.1 Intestinal epithelial cell mitochondrial dysfunction and alteration of IEC renewal and apoptosis

As described earlier, alterations of mitochondrial function in IEC can have major consequences on intestinal epithelium homeostasis through enhanced apoptosis or impairment of ISC metabolism and proliferation. This applies also to HFD-induced mitochondrial dysfunction. Indeed, parallel to the oxidative stress and mitochondrial alterations observed in colonocytes of mice fed a HFD for 12 weeks, an increase by 50% of p53 upregulated modulator of apoptosis (PUMA) expression was observed, although no other apoptotic markers have been studied [134]. Likewise, HCT-116 cells treated for 24 h with 100 μM palmitic acid exhibited hallmarks of apoptosis, characterized by increased protein expression of cleaved Caspase-3 and PUMA (Table 2).

High fat diet consumption has also been shown to impact ISC activity through a mechanism potentially involving the mitochondria. Crypts extracted from the jejunum of mice fed a HFD (60% kcal fat) for 6 months generated more organoids than those extracted from the jejunum of low fat diet-fed mice (10% kcal fat) [71]. Organoids from HFD-fed mice jejunum also exhibited higher expression of proteins involved in fatty acid β-oxidation, notably through increases in PPARδ and PPARα expressions. This enhanced PPAR-fatty acid β-oxidation program was responsible of stemness enhancement whereas fatty acid β-oxidation impairment, through deletion or inhibition of CPT1, suppressed those effects [71]. Furthermore, in mouse proximal small intestine organoids and *Drosophila,* deletion of the mitochondrial pyruvate carrier, impairing TCA cycle to the benefit of fatty acid β-oxidation, is sufficient to increase ISC proliferation whereas its overexpression suppress it [135]. Even though mitochondrial function has not been precisely evaluated in these models, the involvement of mitochondria is possible since fatty acid β-oxidation is strongly linked to mitochondrial function and ROS generation.

#### 4.2.2 Intestinal epithelial cell mitochondrial dysfunction and disruption of intestinal barrier function

Chronic HFD consumption induces intestinal barrier disruption notably through tight junction restructuring [136]. The exact mechanisms leading to altered barrier function under HFD is still debated and could be explained by tight junction disruption, inflammation, alterations of mucus layer, dysbiosis and oxidative stress linked to fatty acid and bile acids [137]. A role of mitochondrial dysfunction is also conceivable. Indeed, impairment of mitochondrial function in T84 cells by dinitrophenol results in increased superoxide detection and concomitant translocation and internalization of *Escherichia coli* that is corrected when using MitoTEMPO, an antioxidant specifically targeting mtROS [138]. Moreover, in their HFD-induced obesity model marked by increased peroxide detection in mouse colonocytes, suggesting the emergence of oxidative stress, Li et al. observed an increased *in vivo* intestinal permeability [134]. Moreover, expression of the tight junction protein occludin was decreased in HFD-fed mouse colonocytes and HCT-116 after palmitic acid treatment [134]. Likewise, hypothesizing that HFD-derived mtROS production participates to intestinal barrier disruption, Watson et al. supplemented their HFD with S3QELs, a suppressor of complex III-derived superoxide. After 16 weeks, mice fed the S3QELs-supplemented HFD were protected against increased *in vivo* intestinal permeability and decreased expression of genes encoding tight junction proteins in colonocytes compared to non-supplemented mice [139].

Another feature of HFD-induced alteration of intestinal barrier function is the reduction of several secreted factors involved in defense mechanisms, such as expression of antimicrobial peptides [140,141]. The α-subunit of hypoxia-inducible factor (HIF) interacts with the β-subunit of HIF and both act as O_2_-sensors. Those transcriptional factors target, among others, genes involved in production of antimicrobial peptides, such as human β defensin-1 [142], intestinal trefoil factor peptides [143] and mucin-3 [144], as well as in creatine metabolism, which appears to be essential in epithelial barrier function [145]. Since OXPHOS requires high amount of oxygen, it participates to the physiological hypoxia observed in healthy colon and small intestine along the crypt-villus axis, from the highest O_2_ pressure in the crypts to the lowest at the top of the villi. Thus, HFD-induced alteration of mitochondrial function and subsequent possible changes in hypoxia level could participate to the altered defense mechanisms observed under HFD through altered HIF signaling.

#### 4.2.3 Intestinal epithelial cell mitochondrial dysfunction and dysbiosis

Dysbiosis in diet-induced obesity has been thoroughly described and incriminated in metabolic alterations in obesity [7]. The relationship between O_2_ level and microbiota composition has already been described using broad-spectrum antibiotics in mice. The loss of epithelial hypoxia and destabilization of HIF expression promote the expansion of facultative anaerobic bacteria, like Enterobactericeae such as *E. coli,* concomitantly with the inhibition of obligate anaerobe growth, including butyrate-producers [64,146]. Recently, Yoo et al. showed that this also applies to chronic HFD consumption. Indeed, HFD-induced mitochondrial bioenergetics defects lead to increased oxygen bioavailability in the colonic lumen favoring the expansion of facultative anaerobic bacteria such as Enterobacteriaceae. Treatment with 5-ASA to increase mitochondrial bioenergetics ameliorated epithelial hypoxia and reduced the fitness advantage of *E. coli* in HFD-fed mice [122]. Moreover, by stimulating the increase in mitochondria number and respiratory capacity (shown by greater cytochrome c oxidase activity), PGC1α improves the aerobic energy production thus favoring epithelial hypoxia and development of obligate anaerobic bacteria [143].

## 5 Conclusion

**Figure 5:**
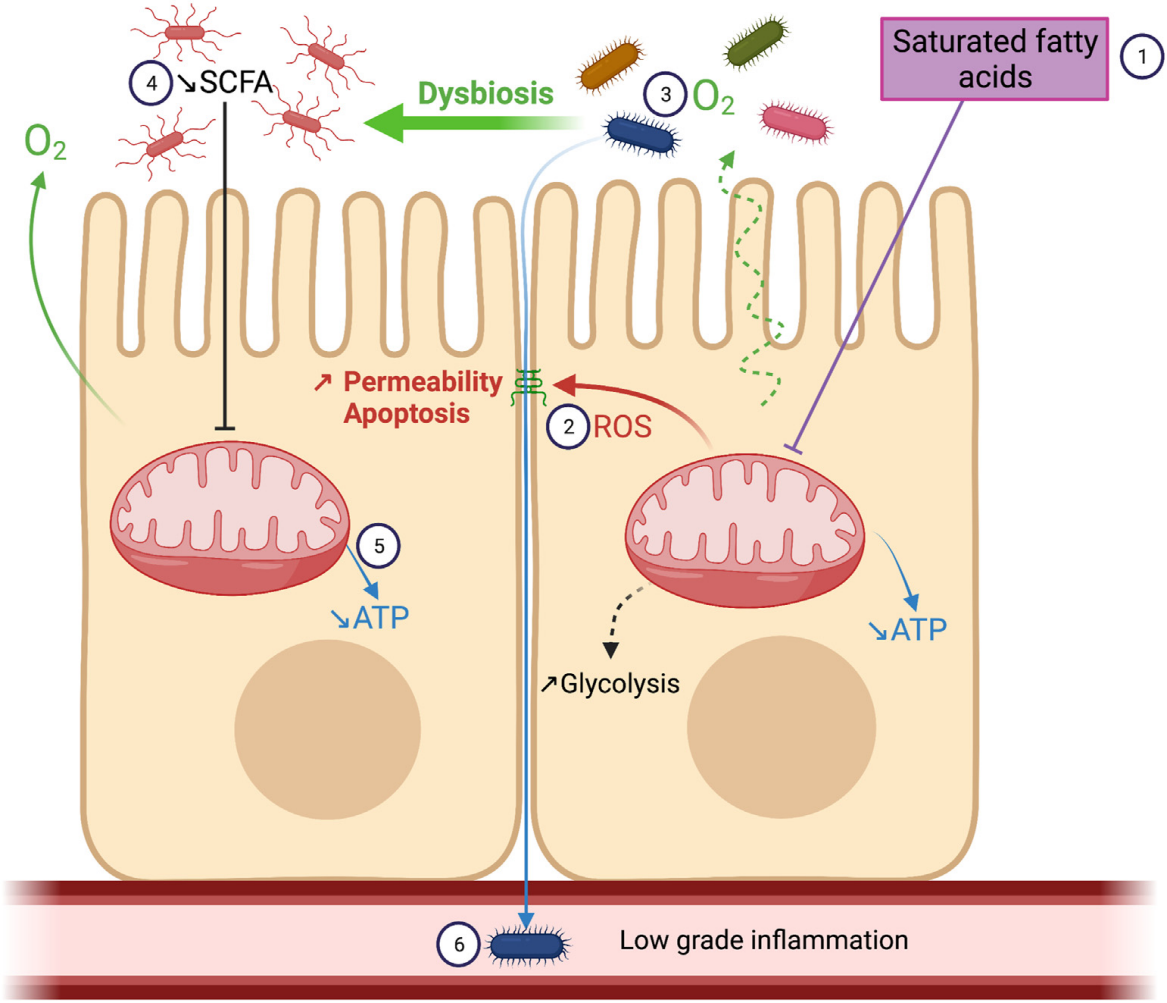
**Proposed mechanisms of mitochondrial dysfunction of colonocytes induced by saturated fatty acids from diet. ①** High fat diet consumption induces mitochondrial dysfunction in colonocytes, possibly mediated by fatty acid metabolism, although this can be debated, and characterized by perturbations of mitochondrial dynamics and shape (swollen mitochondria), and decreased ATP production as a consequence of altered OXPHOS activities. **②** Mitochondrial dysfunction is associated with the emergence of oxidative stress which may generate epithelial barrier alterations through tight junction restructuring and IEC apoptosis, which could increase intestinal permeability. **③** Decreased OXPHOS levels is linked to O_2_ leaks into the intestinal lumen, inducing **④** loss of epithelial hypoxia, colonic dysbiosis and decreased short**-**chain fatty acid (SCFA) concentrations, including butyrate, the preferred energy fuel for colonocytes, leading to decreased levels of ATP. **⑤** Taken together, those alterations could promote bacterial translocation and systemic low-grade inflammation.

Because of the high amount of energy required to ensure rapid epithelial turnover, nutrient transport and maintenance of epithelial barrier, intestinal epithelium homeostasis largely depends on mitochondrial activity, notably via OXPHOS. Moreover, as IEC migrate upward the crypt-villus axis, they encounter different metabolic states associated with different mitochondrial activities due to metabolic cooperation which ensures epithelial homeostasis. To fuel energy production, metabolites provided from the diet or bacterial fermentation are used by IEC as substrates. Yet, dietary changes, and more particularly HFD consumption, seem to modulate mitochondrial function, as recently described in colonocytes. However, the mechanisms involved in these defects have not been described yet, although several authors hypothesized a role of dietary fatty acid β-oxidation (Figure 5). In addition, mitochondrial function in IEC in response to lipid challenge is not clear in the small intestine. Data indicate that enhanced fatty acid catabolism could be a mechanism that compensates lipid storage in enterocyte and ameliorates glucose homeostasis and reduces fat gain. Yet, although fatty acid β-oxidation is increased few days after HFD consumption, it is still unclear how this catabolic pathway evolves with longer periods of HFD consumption and how it affects mitochondrial function of small intestine epithelial cells. Nevertheless, excessive fatty acid oxidation may aggravate HFD-induced oxidative stress which may in turn alter epithelial barrier, favoring low grade inflammation and may promote tumorigenicity. Getting a better understanding of mitochondrial function in IEC under HFD would help to identify mitochondrial target/regulators in order to prevent intestinal alterations and the emergence of metabolic disorders.
